# The role of inflammasome dysregulation in obstructive and non-obstructive azoospermia: a comparative molecular analysis of blood, tissue, and seminal plasma

**DOI:** 10.3389/fimmu.2024.1507885

**Published:** 2024-12-06

**Authors:** Seyyed AmirHossein Mirghanizadeh Bafghi, Farzaneh Fesahat, Fateme Zare, Maryam Imani, Serajoddin Vahidi, Hossein Ansariniya, Ali ZareHoroki, Hossein Hadinedoushan

**Affiliations:** ^1^ Reproductive Immunology Research Center, Shahid Sadoughi University of Medical Sciences, Yazd, Iran; ^2^ Research and Clinical Center for Infertility, Yazd Reproductive Sciences Institute, Shahid Sadoughi University of Medical Sciences, Yazd, Iran; ^3^ Department of Immunology, Faculty of Medicine, Shahid Sadoughi University of Medical Sciences and Health Services, Yazd, Iran

**Keywords:** inflammasome, azoospermia, gene expression, interleukin, seminal plasma

## Abstract

**Background:**

To address knowledge gaps, this study aimed to investigate the involvement of inflammasomes in the etiology of azoospermia. This study focused on the gene expression of key inflammasome components, including *NLR family pyrin domain containing 3 (NLRP-3), CASPASE-1, Interleukin-1β (IL-1β), Interleukin-18 (IL-18), NLR family CARD domain-containing protein 4/ice protease-activating factor (NLRC-4/IPAF)*, and *Absent in melanoma 2 (AIM-2)*.

**Methods:**

We analyzed gene expression in blood and testicular tissue from patients with obstructive azoospermia (OA) and non-obstructive azoospermia (NOA). Additionally, we compared IL-1β and IL-18 expression levels in seminal plasma samples using the Enzyme-Linked Immunosorbent Assay (ELISA) method. For comparison, blood samples from normospermic (NS) individuals were also genetically evaluated.

**Results:**

Our results indicated significantly higher gene expression of inflammasome components in NOA patients than those in OA patients either in blood or in testicular tissue. Both azoospermic groups exhibited higher mRNA levels of inflammasome genes comparing with those from blood samples of NS men. Seminal plasma samples showed significantly increased levels of IL-1β and IL-18 in NOA patients compared to men with OA. The ROC curve analysis indicated strong and significant predictive power of *IL-18, AIM-2* and *NLRC-4/IPAF* gene expression profiles between NOA vs. NS patients and NOA vs. OA.

**Conclusions:**

Our findings highlight the role of hidden chronic inflammation in azoospermia, particularly within the NOA group. This study provides a foundation for further detailed research, which could aid in the development of diagnostic panels to differentiate between various azoospermic groups.

## 1 Introduction

Azoospermia, characterized by the absence of spermatozoa in the seminal fluid, affects 5 to 20 percent of infertile men, compared to approximately 2 percent of the general population of reproductive age (1, 2). It arises when males are unable to produce spermatozoa due to various factors, or when there is an obstruction in the passage of spermatozoa from the testes to the ejaculatory ducts (3). The causes of azoospermia can be classified into three categories: pre-testicular causes (secondary testicular failure), testicular causes (primary testicular failure), and post-testicular causes (obstruction) (4). However, in clinical practice and treatment decision-making, azoospermia is often categorized into two main types: obstructive azoospermia (OA) and non-obstructive azoospermia (NOA) (5). OA can result from various factors, including genetic diseases, vasectomy, congenital absence of the vas deferens, and idiopathic or acquired epididymal obstruction, such as sexually transmitted infections (6). Similarly, NOA can be attributed to a wide range of causes, including genetic/chromosomal abnormalities, iatrogenic/surgical interventions, developmental/structural etiologies, and hypogonadism. Despite advances in understanding the underlying conditions that lead to azoospermia, a significant proportion of cases remain idiopathic (7).

Emerging evidence suggests that chronic inflammation may contribute to idiopathic azoospermia, both OA and NOA. Immunological factors are known to play a significant role in inflammatory processes and contribute to idiopathic azoospermia (8). The inflammasome, a crucial structure involved in inflammation, has been implicated in certain cases of azoospermia, such as varicocele (9, 10). The importance of the inflammasome in male infertility is increasingly recognized, with *NLRP-3* and *IL-1* being identified as regulators of spermatogenesis. Their effects extend to enhancing various conditions associated with male infertility, highlighting the role of inflammasomes in the immune response within the testes, blood, seminal plasma, etc. (11, 12).

The inflammasome, a component of innate immunity, is triggered by exposure to viruses, bacteria, fungi, and non-organic stimuli. Key components of the inflammasome include *NLRP-3*, *CASPASE-1*, *IL-1β*, *IL-18*, *AIM-2*, and *NLRC4/IPAF*. When *NLRP-3*, *AIM-2*, and *NLRC-4/IPAF* are activated, they trigger the conversion of *pro-CASPASE-1*, *pro-IL-1β*, and *pro-IL-18* into their active forms, *CASPASE-1*, *IL-1β*, and *IL-18*, respectively (8, 13, 14). NLRP-3 have been implicated as a central player in the immune response of the male reproductive system. When testicular inflammation occurs, cells can release pro-inflammatory cytokines like IL-1β and CCL2 via a mechanism requiring the NLRP-3 inflammasome. Previous studies have reported elevated concentrations of crucial inflammasome components in the semen of men with reproductive diseases, suggesting a potential link between inflammasome activation and disease pathogenesis (15, 16).

Strong inflammasome activation preferentially leads to caspase 1 recruitment and activation, IL-1β and IL-18 activation, and inflammation. Weak inflammasome activation promotes ASC-mediated caspase 8 recruitment and activation of the apoptotic pathway (17). Caspase 8 may play a role in the infertility caused by long-term alcohol consumption (18). Mature *IL-1β* and *IL-18* are produced by macrophages and dendritic cells in response to different types of cellular stress (19, 20). The Caspases have been implicated in the pathogenesis of multiple andrological pathologies, such as impaired spermatogenesis, decreased sperm motility and increased levels of sperm DNA fragmentation, testicular torsion, varicocele, and immunological infertility (21).

**Figure 1:**
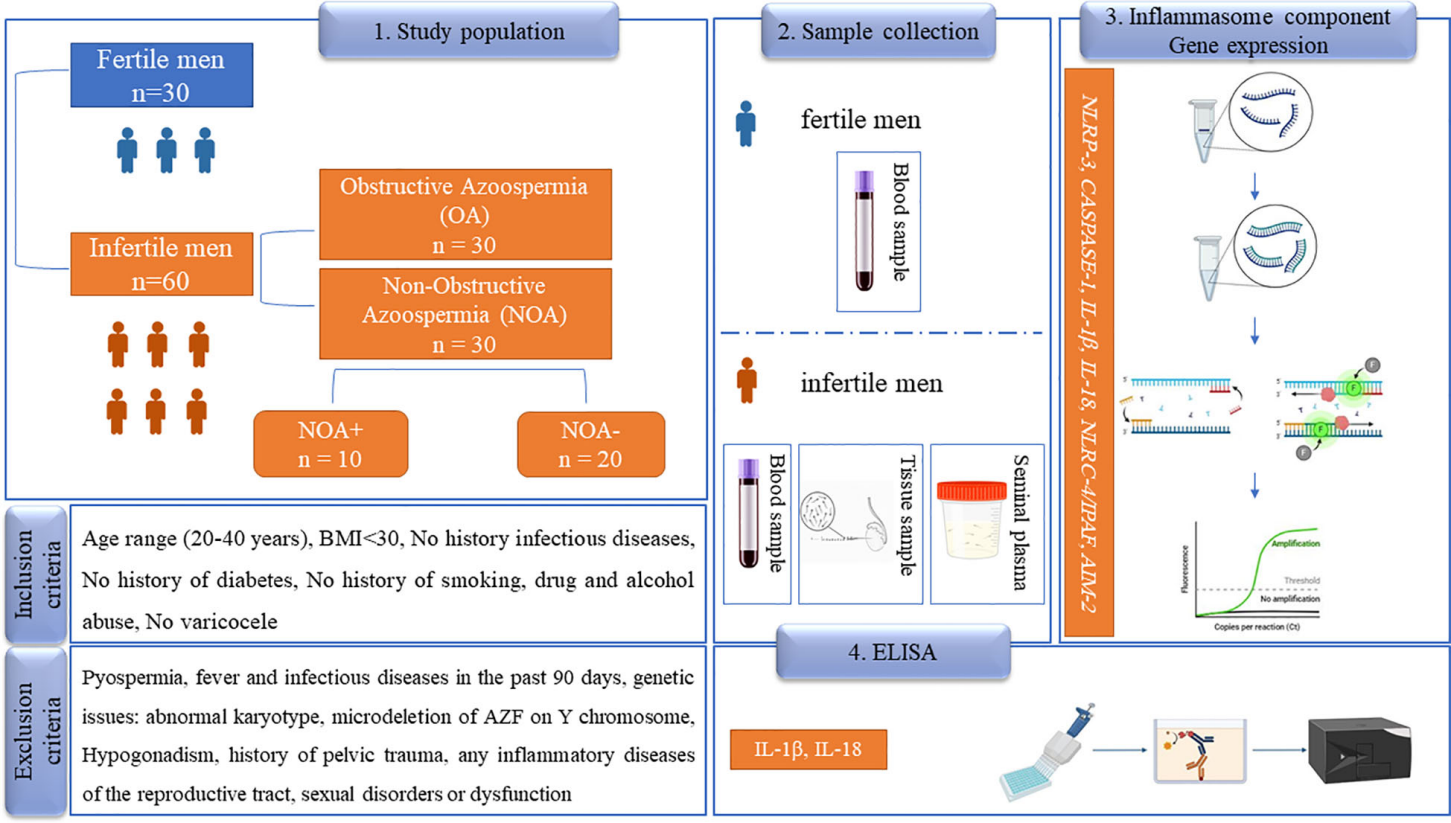
The study design of the paper. In this prospective study, 60 patients with azoospermia were examined, while 30 healthy, fertile men served as a control group (normospermia). OA, obstructive azoospermia; NOA, non-obstructive azoospermia; NOA+, successful sperm retrieval; NOA-, unsuccessful sperm retrieval; BMI, body mass index; AZF, azoospermia factor; *NLRP-3, NLR family pyrin domain containing 3; NLRC4/IPAF, NLR family CARD domain-containing protein 4/ ice* *protease-activating factor, AIM-2, absent in melanoma;* *2 IL-1β, interleukin-1β; IL-18, interleukin-18*; ELISA, Enzyme-Linked Immunosorbent Assay. The pictures are taken from the Biorender website.

Despite the growing interest in inflammasomes, the literature currently lacks compelling evidence regarding their specific role in azoospermia. This research gap motivated the primary goal of this study investigating the involvement of inflammasomes in the etiology of azoospermia. The study design is shown schematically in **Figure 1**.

## 2 Materials and methods

### 2.1 Study population

Participants were divided into two main groups: patients with OA and infertile men with NOA. Each group consisted of 30 patients. The NOA group was further subdivided into subgroups based on sperm retrieval outcomes: NOA+ (successful sperm retrieval) and NOA- (unsuccessful sperm retrieval). Additionally, 30 healthy fertile men served as a control group, referred to as normospermia (NS), for comparison of blood outcomes. Inclusion criteria for the NS group included normospermic sperm cells and at least one child within the past 2 years. Demographic variables, including age, duration of infertility, and occupation, were collected through patient questionnaires and face-to-face interviews with all participants. For the NOA and OA groups, men were selected based on the following criteria: primary infertility, age between 20-40 years, body mass index (BMI) below 30, no history of smoking or drug abuse, no diabetes, no alcohol consumption, and no varicocele. Patients with pyospermia (presence of more than one million white blood cells per milliliter of semen), fever or infectious diseases within the past 90 days, abnormal karyotype, microdeletion of the AZF region on the Y chromosome, hypogonadism, abnormal levels of sexual hormones (prolactin/FSH/testosterone), any infections and/or inflammatory diseases related to the reproductive tract, and sexual disorders or dysfunction in ejaculation were excluded from the study population.

### 2.2 Measurement of IL-1β and IL-18 in seminal plasma

Quantitative assessments of IL-1β and IL-18 concentrations in seminal plasma were performed using ELISA Kits (R&D, USA) according to the manufacturer’s instructions. Semen samples were collected from both NOA and OA groups after at least 72 hours of the last ejaculation, and seminal plasma was subsequently isolated. The sensitivities of the IL-1β and IL-18 assays were 1.0 pg/ml and 5.15 pg/ml, respectively.

### 2.3 Gene expression assessments

#### 2.3.1 Sample collection

Blood samples and testicular biopsy samples were collected for gene expression analysis. Testicular biopsy samples were obtained from NOA patients using micro-TESE (Testicular Sperm Extraction) and TESA (Testicular Sperm Aspiration), while OA patients underwent TESE/PESA (Percutaneous Epididymal Sperm Aspiration). Following testicular biopsy, sperm presence and analysis were conducted in the andrology laboratory for each patient. Due to ethical considerations, only blood samples were collected from fertile participants. Blood samples (from the antecubital vein) were collected from all study groups between 8:00 AM and 11:00 AM.

#### 2.3.2 RNA extraction and cDNA synthesis

Total RNA was extracted from various tissue and blood samples using a total RNA extraction kit from SinaclonBioscience, Iran, following the manufacturer’s instructions. The purity and concentration of each extracted RNA sample were measured using a spectrophotometer at optical densities of 260/280 and 260 nm, respectively. RNA samples with values ranging from 1.8 to 2.0 were normalized prior to cDNA synthesis (22). Subsequently, cDNA synthesis for each RNA sample was performed using the Revert Aid First Strand cDNA Synthesis Kit, following the protocol provided by Parstous Biotechnology, Iran. The synthesized cDNA was stored at -20°C until use.

#### 2.3.3 Quantitative real-time PCR

Real-time quantitative reverse transcriptase-polymerase chain reaction (qRT-PCR) was conducted using specific primers for inflammasome components (*NLRP-3*, *CASPASE-1*, *IL-1β*, *IL-18*, *NLRC-4/IPAF*, *AIM-2*) and *GAPDH* as a housekeeping gene (**Table 1**) along with SYBR green master mix (Parstous, Iran). All specific primers used in this study have been selected after deep search in data resources and papers published in global and valid journals. Specificity and accuracy of each primer has been verified using Primer-BLAST tool. Prior to start the main qPCR run, each primer has been checked and set up for optimal Tm and concentrations, and efficiently. All primers with efficiently more than 95% considered for perform qPCR using the 2-△△CT method. During each run, melt curve stage has been considered along with run method due to verify the specificity of own primer. Each qRT-PCR reaction consisted of 1 µL cDNA, 0.5 µL forward primer, 0.5 µL reverse primer, 5 µL master mix, and 3 µL nuclease-free water. The reactions were performed in duplicate using the StepOne system from Applied Biosystems, CA, USA. The qRT-PCR protocol involved an initial denaturation step (15 minutes at 95°C) followed by 40 cycles of amplification at 95°C for 15 seconds, 58°C, 60°C, or 64°C (depending on the specific primer) for 30 seconds, and 72°C for 30 seconds. A final extension step was conducted at 72°C for 5 minutes, followed by a melting curve analysis. Data analysis for relative gene expression was performed using the 2^-△△CT^ method.

**Table 1:** Oligonucleotide primer sequences used in this study.

Genes	Primer Sequence (5´-3´)	Accession No.	PCR Product (bp)
*NLRP-3*	F: GGAGTGGATGGGTTTACTGGAG R: CGTGTGTAGCGTTTGTTGAGG	NM_001079821.3	165
*CASPASE-1*	F: TGAATACCAAGAACTGCCCAAG R: GCATCATCCTCAAACTCTTCTGTAG	NM_001257118.3	157
*IL-1β*	F: AGCTCGCCAGTGAAATGATG R: TGTAGTGGTGGTCGGAGATT	NM_000576.3	156
*IL-18*	F: TCTTCATTGACCAAGGAAATCGG R: TCCGGGGTGCATTATCTCTAC	NM_001386420.1	75
*AIM-2*	F: TCAAGCTGAAATGAGTCCTGC R: CTTGGGTCTCAAACGTGAAGG	NM_001348247.2	206
*NLRC-4/IPAF*	F: TGAACTGATCGACAGGATGAAC R: GTCTCCAGTTTTTCAACCCAAG	NM_001199138.2	149
*GAPDH*	F: AAATCAAGTGGGGCGATGCTG R: GCAGAGATGATGACCCTTTTG	NM_001256799.3	118

PCR, Polymerase chain reaction; NLRP-3, NLR family pyrin domain containing 3; IL-1, interleukin-1; IL-18, interleukin-18; AIM-2, absent in melanoma 2; NLRC4/IPAF, NLR family CARD domain-containing protein 4 and GAPDH, Glyceraldehyde-3-phosphate dehydrogenase.

### 2.4 Statistical analysis

The sample size for this study was determined using statistical formulas and the Morgan table (α=0.05, β=0.2). Statistical analyses were conducted using GraphPad Prism software and SPSS 24 for Windows. Data were presented as means ± Standard Error of the Mean (SEM). The paired t-test or Mann–Whitney test was chosen based on data normality, which was assessed using the Kolmogorov-Smirnov test. One-way ANOVA (and nonparametric or mixed models) were used for specific group comparisons. Dunn’s Test was performed as a *post-hoc* analysis following a one-way ANOVA when significant differences were detected among multiple groups. Additionally, the Pearson correlation test was employed to evaluate the correlation between variables. A P-value less than 0.05 was considered statistically significant.

## 3 Results

**Table 2** shows that there were no significant differences between the NS, NOA, and OA groups in terms of mean age and BMI. Additionally, no significant differences were observed in any of the sexual hormone levels among the study groups. Within the NOA group, 20 patients (66.6%) had unsuccessful sperm retrieval, while 10 patients (33.3%) had successful sperm retrieval.

**Table 2:** The baseline demographic and laboratory indicators of the study participants.

Groups Variables	Normospermia	Obstructive azoospermia	Non-obstructive azoospermia	P-value
Age (Year)	32.15 ± 1.215	35.38 ± 0.82	36.29 ± 1.27	ns
BMI (Kg/m^2^)	26.70 ± 0.40	26.20 ± 0.37	26.58 ± 0.32	ns
FSH (IU/L)	7.01 ± 0.59	6.27 ± 0.47	8.21 ± 0.81	ns
LH (IU/L)	6.1 ± 0.37	5.94 ± 0.32	6.25 ± 0.46	ns
Testosterone (ng/ml)	5.7 ± 0.57	4.30 ± 0.31	6.7 ± 2.0	ns
Prolactin (ng/ml)	10.78 ± 0.7	11.28 ± 0.73	10.32 ± 0.65	ns

FSH, follicle-stimulating hormone; LH, luteinizing hormone; NS, Not significant data. Data were presented as Mean ± SEM. P ≥ 0.05 is not considered significant.

### 3.1 Gene expression profile in blood samples

**Figure 2:**
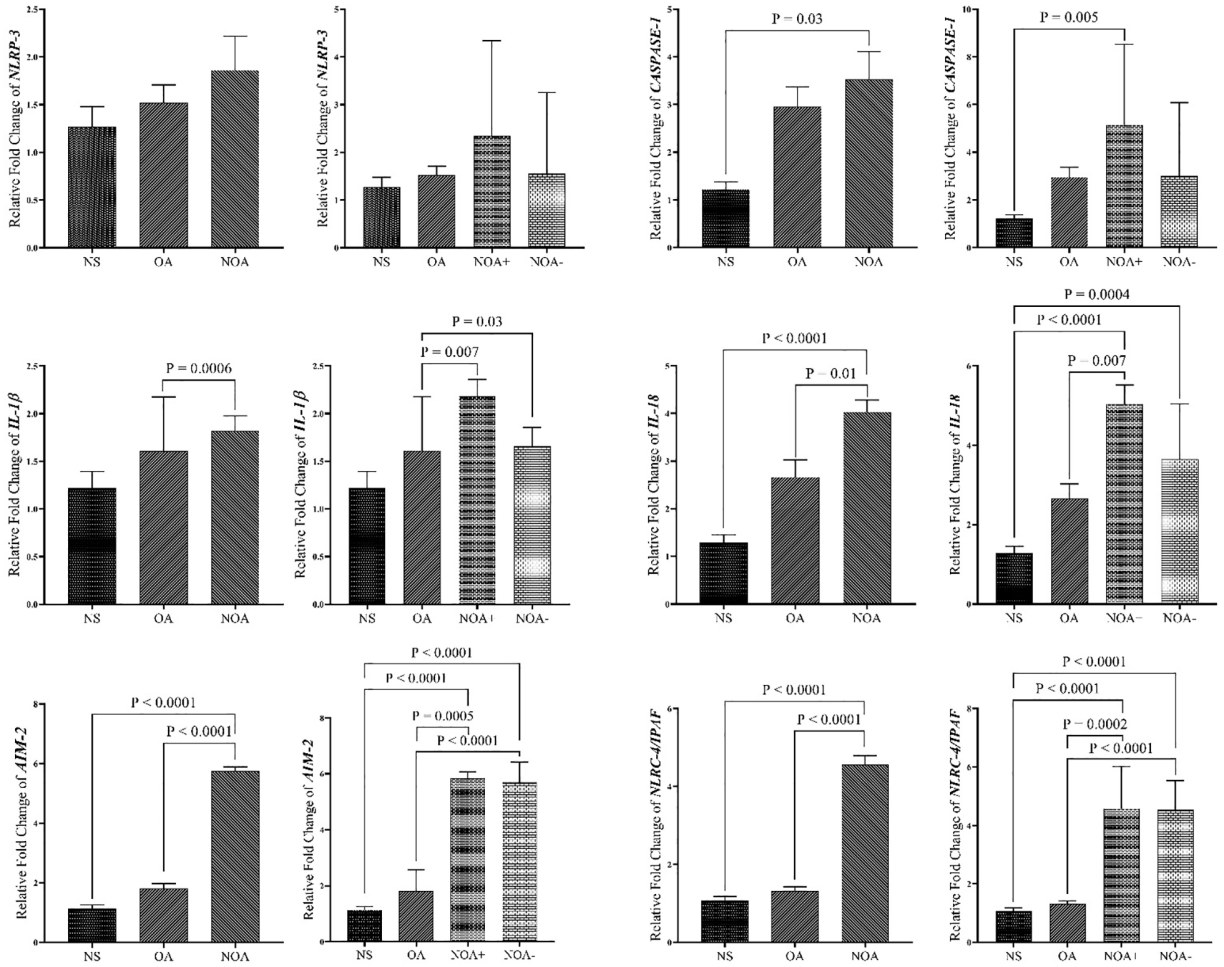
Comparison of gene expression in blood samples of study groups. The data are presented as mean ± SEM, performed by One-way ANOVA test. P < 0.05 represents significant values. NS, normospermia; OA, obstructive azoospermia; NOA+, positive sperm retrieval non-obstructive azoospermia; NOA-, negative sperm retrieval nonobstructive azoospermia; *NLRP-3, NLR family pyrin domain containing 3; NLRC4/IPAF, NLR family CARD domain-containing protein 4/ ice* *protease-activating factor; AIM-2, absent in melanoma* *2; IL-1β, interleukin-1β; IL-18, interleukin-18*.

Across all studied genes, the highest expression levels were observed in the NOA group compared to other groups. Conversely, the lowest mRNA levels for all genes were found in the NS group. Our data revealed that *CASPASE-1, IL-18, AIM-2*, and *IPAF/NLRC-4* genes were significantly overexpressed in the NOA group, as well as the NOA+ subgroup, compared to the NS group. *IL-1β, IL-18, AIM-2*, and *NLRC-4/IPAF mRNA* levels were significantly higher in the NOA group compared to the OA group. No significant differences were observed between the NS group and the OA group for any of the target genes. Additionally, no significant differences in *NLRP-3* gene expression were found between any of the groups. While the NOA+ subgroup exhibited higher expression levels for all genes, no significant changes were detected between the two NOA subgroups. There was a significant upregulation in *IL-1β, IL-18, AIM-2*, and *NLRC4/IPAF* genes in the NOA+ subgroup compared to OA cases (**Figure 2**).

**Figure 3:**
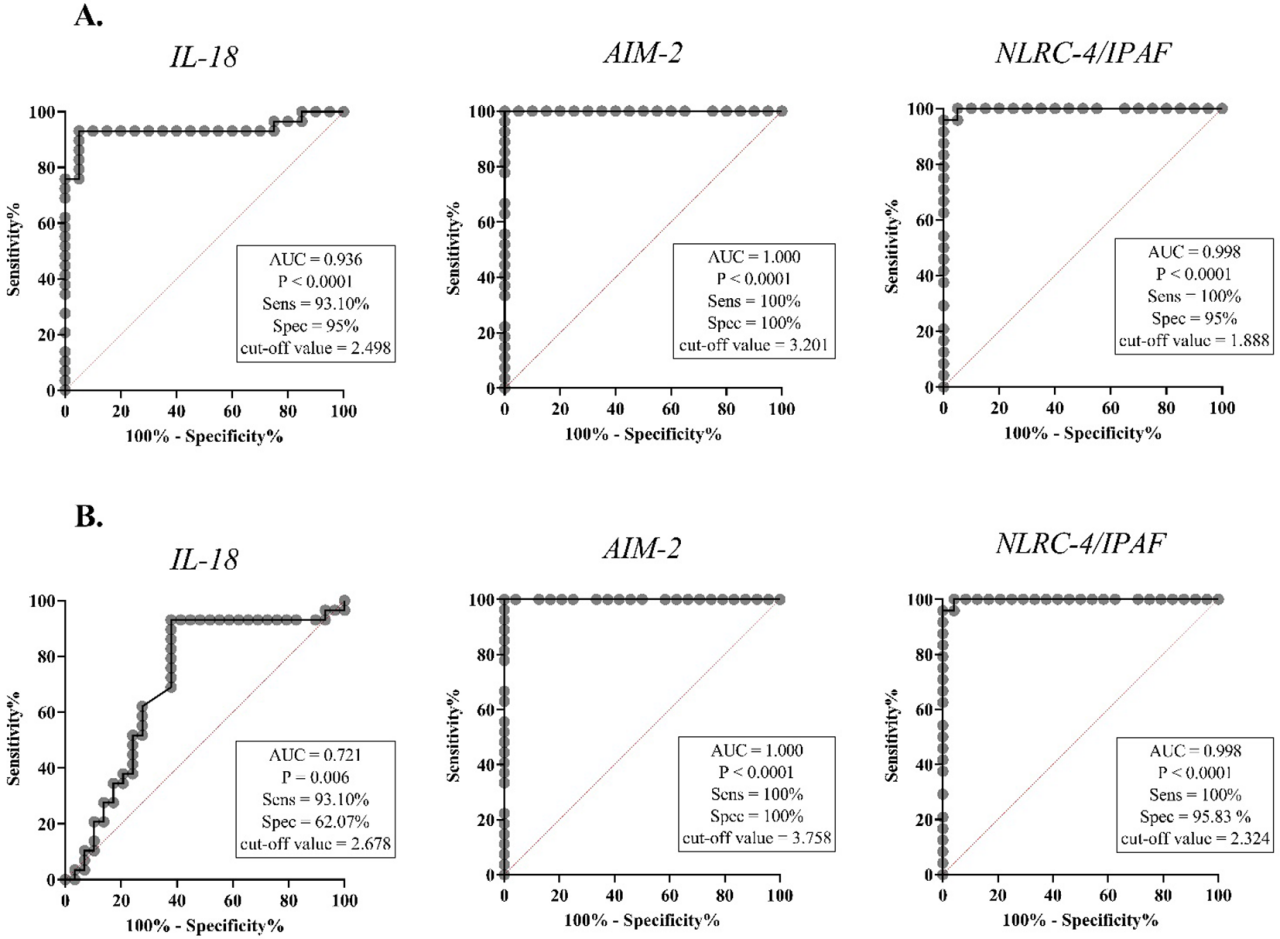
The receiver operating characteristic (ROC) analysis of *IL-18, AIM-2, and NLRC-4/IPAF* mRNA levels in blood samples. The right-hand panel of each graph displays the area under the ROC curve (AUC), the optimal cut-off value determined by the Youden index, and the corresponding sensitivity and specificity values for each prediction. IL-18, interleukin-18; AIM-2, absent in melanoma 2; NLRC4/IPAF, NLR family CARD domain-containing protein 4/ ice protease-activating factor. **(A)** ROC curve comparing NOA and NS in blood samples, **(B)** ROC curve comparing NOA and OA in blood samples. P < 0.05 is regarded as a significant value. AUC, area under curve; Sens, sensitivity; Spec, specificity.

ROC curve analysis was performed for all target genes. The ROC curve analysis indicated strong and significant predictive power of *IL-18, AIM-2*, and *NLRC-4/IPAF* gene expressions in differentiating between NOA vs. NS patients and NOA vs. OA infertile men (**Figure 3**). *IL-18* gene expression exhibited a sensitivity of 93.10%, specificity of 95%, and area under the curve (AUC) of 62.07%, with cut-off values (COV) of 2.498 and 2.678 for NOA vs. NS and NOA vs. OA, respectively. *AIM-2* mRNA levels demonstrated a sensitivity and specificity of 100%, with a COV of 3.201 and 3.758 for NOA vs. NS and NOA vs. OA, respectively. When comparing NOA vs. NS and NOA vs. OA, *NLRC-4/IPAF* mRNA levels displayed a sensitivity of 100% and specificity of 95% and 95.83%, respectively, with a COV of 1.888 and 2.324.

### 3.2 Gene expression profile in tissue samples

**Figure 4:**
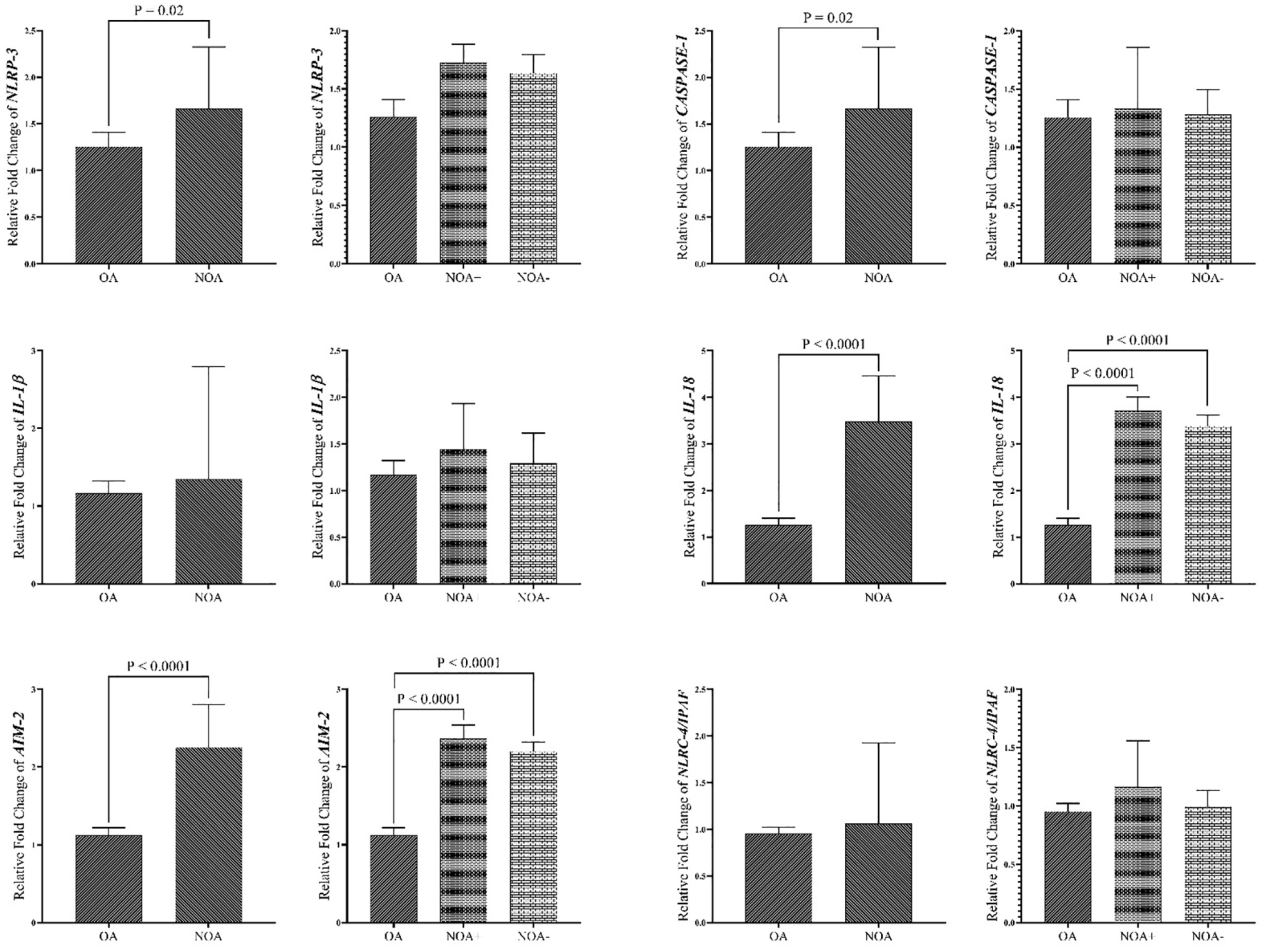
Comparison of Gene expression in tissue samples of study groups. The data are presented as mean ± SEM , performed by paired t-test or Mann–Whitney test was chosen based on data normality and One-way ANOVA test. P < 0.05 represents significant values. OA, obstructive azoospermia; NOA, non-obstructive azoospermia; NOA, non-obstructive azoospermia; NOA+, positive sperm retrieval nonobstructive azoospermia; NOA-, negative sperm retrieval non-obstructive azoospermia; NLRP-3, NLR family pyrin domain containing 3; NLRC4/IPAF, NLR family CARD domain-containing protein 4/ ice protease-activating factor; AIM-2, absent in melanoma 2; IL-1β, interleukin-1β; IL-18, interleukin-18.

**Figure 4** demonstrates that overexpression of all genes was more pronounced in the NOA group compared to the OA group. When comparing the two NOA subgroups, overexpression was observed to be higher in the NOA+ group compared to the NOA- group. *NLRP-3*, *IL-18*, and *AIM-2* gene upregulation were significantly higher in the NOA group compared to the OA group. *AIM-2* and *IL-18* expression levels were significantly higher in both the NOA+ and NOA- groups compared to the OA group. No significant differences were observed between the NOA+ and NOA- groups. In both study groups, ROC curve analysis of *IL-18* and *AIM-2* demonstrated a strong and significant predictive capacity in all azoospermic men (sensitivity of 85.19% vs. 83.87%, specificity of 100% vs. 83.87%, COV of 2.329 vs. 1.772, respectively). However, no strong ROC values were observed for NLRC-4/IPAF against blood data (**Supplementary Figure 1**).

### 3.3 Concentration of IL-1β and IL-18 in seminal plasma samples

Protein levels of *IL-1β* and *IL-18* were significantly higher in the NOA group compared to the OA group (p=0.0036) (**Table 3**). **Table 4** reveals a positive and significant correlation between these two cytokines in both groups of azoospermia.

**Table 3:** Interleukin levels of seminal plasma in azoospermic samples.

Groups Cytokines	Obstructive azoospermia	Non-obstructive azoospermia	P-value
IL-1β	5.03 ± 0.87	12.58 ± 1.57	0.0036*
IL-18	33.18 ± 8.1	108.0 ± 15.7	0.0036*

IL-1β, interleukin-1; IL-18, interleukin-18. Data were presented as Mean ± SEM. P < 0.05 represents significant values.

**Table 4:** Correlation between seminal plasma concentrations of IL-1β and those in IL-18.

	Variables	Interleukin 1 β		Interleukin 18	
Variables	Groups	Obstructive azoospermia	Non-obstructive azoospermia	Obstructive azoospermia	Non-obstructive azoospermia
**Interleukin 1β**	Obstructive azoospermia	1 -	-0.67 0.15	0.83 0.04*	**-0.68** **0.13**
	Non-obstructive azoospermia	-0.67 0.15	1 -	-0.25 0.64	**0.88** 0.001*
**Interleukin 18**	Obstructive azoospermia	0.83 0.04*	-0.25 0.64	1 -	**-0.44** **0.38**
	Non-obstructive azoospermia	-0.68	0.88	-0.44	**1**
		0.13	0.001*	0.38	**-**

The Pearson and Spearman correlation tests were used. In each cell, the top value is correlation coefficient (r ) and the bottom is the p value. The P-values < 0.05 were considered to indicate statistical significance. Where P-values are significant, numbers are written in bold and marked by an asterisk.

**Supplementary Figure 2** indicates that no significant correlation was observed between *IL-1β* and *IL-18* in the gene expression of total samples and the levels of seminal plasma cytokines in both groups.

### 3.4 Correlation

**Figure 5:**
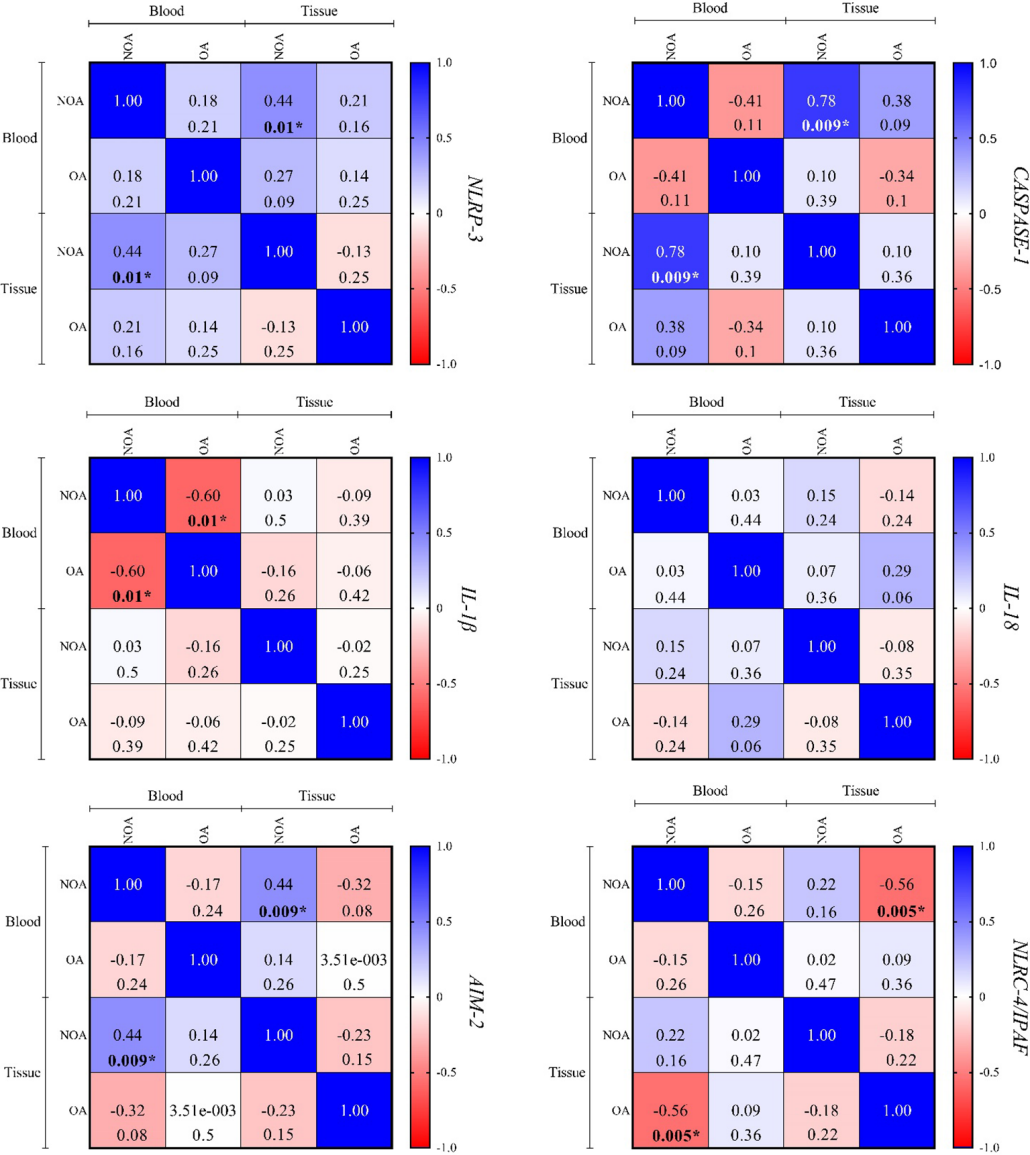
The correlation between the blood and tissue samples was performed using Spearman's rank correlation. In each cell, the top value is correlation coefficient (r), and the bottom is the P-value. P < 0.05 is regarded as a significant value. Where P-values are significant, numbers are written in bold and marked by an asterisk. OA, obstructive azoospermia; NOA, non-obstructive azoospermia; NLRP-3, NLR family pyrin domain containing 3; IL-1β, interleukin-1β; IL-18, interleukin-18; AIM-2, absent in melanoma 2; NLRC4/IPAF, NLR family CARD domain-containing protein 4/ ice protease-activating factor.

**Figure 5** shows a direct and significant correlation between blood and tissue samples for *NLRP-3*, *CASPASE-1*, and *AIM-2* in the NOA group, but no correlation was observed in the OA group. Additionally, no significant correlations were found for the other three genes in either group. Referring to **Figures 3**, **4** in the supplementary data for more information, no specific or significant correlations were observed among all genes in each group.

## 4 Discussion

Inflammasomes are cytoplasmic multiprotein complexes composed of inflammatory caspases, a sensor protein, and, depending on the specific context, an adapter protein that links the two. These complexes can be activated by a diverse range of endogenous and exogenous stimuli, leading to the enzymatic activation of canonical *CASPASE-1*. This activation results in the secretion of *IL-1β* and *IL-18*, as well as apoptotic and pyroptotic cell death (23).

A deeper understanding of the molecular mechanisms underlying *NLRP-3* inflammasome activation will provide opportunities to develop strategies for preventing and treating diseases, including male infertility associated with the *NLRP-3* inflammasome (24). The first evidence of inflammasome complex involvement in an infertility-related disease was reported in a prospective study in 2013 by Zheng et al. This study found that inflammasome complex proteins were activated in men with spinal cord injury, and their results indicated increased seminal plasma concentrations of *IL-1β* and *IL-18* compared to a control group (25).

Our data demonstrated that the expression of all inflammasome-associated genes was higher in the group with NOA followed by the group with OA, compared to NS. Furthermore, within the NOA subtype, NOA+ exhibited higher expression levels than NOA-. A cross-sectional cohort study conducted by Ordek et al. in 2023 yielded similar results to our findings. *NLRP-3* and *IL-1β* levels were measured in serum and semen. Their study found that serum and seminal plasma *NLRP-3* and *IL-1β* levels in patients with varicocele or azoospermia were significantly higher than in those without either condition (26). In a 2022 study by Kati et al., examining the function of *NLRP-3* and *IL-1β* markers in patients with TESE+ and TESE-, they observed increased expression of these two genes in the TESE+ group compared to TESE-, which was inconsistent with our findings (27). Malcher et al. (2013) reported significantly downregulated *IL1-RA* gene expression in the infertile group compared to controls, while expression levels of *IL-1a* and *IL-1b* did not show significant differences between groups. They also observed significantly higher expression levels of the *CASPASE-1* gene in the Sertoli-cell-only syndrome group (6). In a 2013 study by Shen et al., increased overexpression of *IL-1β* and *IL-18* was found to be associated with an increased number of abnormal sperm cells, reduced sperm motility, and ultimately, infertility (28). Ata-abadi et al. examined varicocele by analyzing semen samples six months before and six months after varicocelectomy. The levels of *IL-1β*, *IL-18*, and *CASPASE-1* cytokines were examined, revealing that following treatment, sperm morphology improved and inflammation in the seminal plasma decreased (29). Inflammasome activation and subsequent overexpression of IL-1β and IL-18 negatively impact spermatogenesis by causing DNA damage, mitochondrial dysfunction, and reduced sperm motility. These effects are likely mediated by the disruption of the seminiferous tubule epithelium and increased germ cell apoptosis (15, 16, 30).

Differences in gene expression affect NOA by described mechanisms. The NLRP**-**3 inflammasome is a multiprotein complex composed of the sensor NLRP-3, the adaptor ASC (apoptosis-associated speck-like protein containing a CARD), and the effector CASPASE-1. This complex facilitates the processing of pro-inflammatory cytokines IL-1β and IL-18 and induces pyroptosis through the cleavage of gasdermin D (15).

Overexpression of the canonical inflammasome gene leads to an increase in ASC. ASC affects multiple sperm structures, including the acrosome, equatorial segment, and midpiece, potentially altering their functions (25).

Elevated *IL-18* levels in semen have been positively correlated with a higher probability of pregnancy failure following IVF and ICSI, suggesting an association with impaired sperm function (31, 32). Current literature provides limited information regarding the role of *AIM-2* and *NLRC-4/IPAF* genes in inflammatory conditions. Onodi et al., conducted a groundbreaking study in 2021, demonstrating how *AIM-2* and *NLRC-4/IPAF* inflammasome activation contributes to persistent inflammation in heart failure (33). Another recent study suggested that overexpression of the *NLRC-4/IPAF* inflammasome could serve as a potential therapeutic target and diagnostic marker for gliomas, potentially associated with poor prognosis (34). Our study’s ROC curve analysis identified three genes, *AIM-2*, *NLRC-4/IPAF*, and *IL-18*, with high sensitivity and specificity in the blood of NOA patients compared to the NS group. In particular view, ROC curve analysis showed that both AIM-2 and IL-18 have significant predictive ability. AIM-2 showed 100% sensitivity and specificity in distinguishing between NOA and NS groups, as well as between NOA and obstructive azoospermia. Similarly, IL-18 showed a sensitivity of 93.10% and a specificity of 95%, confirming its utility as a reliable biomarker. The insights gained from the study of inflammasome biomarkers, particularly AIM-2 and IL-18, can significantly inform future research and clinical practices. Firstly, the high sensitivity and specificity of these biomarkers suggest their potential inclusion in diagnostic panels for azoospermia, allowing clinicians to more accurately differentiate between obstructive and non-obstructive forms. Moreover, future research could explore the integration of additional biomarkers identified in recent studies, such as those related to macrophage infiltration in NOA or seminal fluid profiles from patients with oligozoospermia and azoospermia (35, 36). These findings suggest that diverse factors contribute to these reactions, potentially attributed to differences in gene expression, genetic variations, epigenetic modifications, signaling pathways, and stimulatory and inhibitory factors of molecules (37–41). Furthermore, ethical constraints and legal regulations prevented us from performing testicular biopsies on fertile men. Consequently, in similar comparative studies involving two groups of azoospermia, obstructive azoospermia patients have been utilized as the control group in comparison to the NOA group (42, 43).

To our knowledge, this study is the first leading study with specific focus on exploring the role of inflammasome in infertile men with different history of azoospermia as the most severe complications relate to male infertility. Despite a little literature on evaluating the potative function of the some involved inflammasome components (not all members) in patients with varicocele (8, 11, 26, 44, 45), our study provides a novel and detailed exploration of inflammasome members and their mechanisms in azoospermia. Additionally, our results highlight the significant role of chronic inflammation in azoospermia, particularly within the NOA group. Although it is too early to provide a definitive opinion, with only a single-center study and a limited sample size, our study has clearly shown the role of the immunological system and the effect of inflammation in the pathogenesis of azoospermia with unknown causes. Additionally, the future perspective of such a this research could lead to introduce and design an accurate and highly specific hybrid-diagnostic panel aiding experts in the treatment process of azoospermic men to estimate of the success rate of sperm recovery surgeries by using molecular and non-invasive tools. Taken together, our findings in line with newly reports (46) emphasizes lack of current data and need for future studies to refine diagnostic approaches.

## 5 Conclusion

Our findings demonstrated a significant increase in both gene and protein expression of inflammasome components in azoospermic patients, particularly in men with NOA. The alignment of gene expression results from tissue and blood samples, coupled with ROC curve analysis, suggests that investigating *AIM-2*, *NLRC-4/IPAF*, and *IL-18* could serve as three potential biomarkers for predicting and differentiating the causes of azoospermia.

### 5.1 Limitations

Due to ethical concerns and the specific conditions of individuals with normospermia, invasive sampling from their testes could not be possible. Therefore, we could compared only samples from blood of azoospermic patient with fertile ones not at the levels of testicular tissue. One of the other limitations was the relatively small number of samples. Although the multiple sample collection in clinical studies similar to ours meet all criteria is usually difficult and time-consuming, we tried to touch the standard sample size similar to other studies (47, 48). It seems that future studies should conduct with larger populations and gather all data reported from multicenter with meta-analysis.

## Data Availability

The original contributions presented in the study are included in the article/**Supplementary Material**. Further inquiries can be directed to the corresponding author.
