# Supplementary material for: The role of inflammasome dysregulation in obstructive and non-obstructive azoospermia: a comparative molecular analysis of blood, tissue, and seminal plasma
Source: Front Immunol. 2024 Dec 6;15:1507885. doi: 10.3389/fimmu.2024.1507885 (PMC11659152; doi:10.3389/fimmu.2024.1507885)
Supplement: Supplementary file 1 [file DataSheet1.docx]

**The Role of Inflammasome Dysregulation in Obstructive and Non-Obstructive Azoospermia: A Comparative Molecular Analysis of Blood, Tissue, and Seminal Plasma**

**Seyyed AmirHossein Mirghanizadeh Bafghi**

*Reproductive Immunology Research Center, Shahid Sadoughi University of Medical Sciences, Yazd, Iran, Orcid ID: 0000-0001-5373-3038*

**Farzaneh Fesahat**

*Reproductive Immunology Research Center, Shahid Sadoughi University of Medical Sciences, Yazd, Iran, Orcid ID: 0000-0002-3743-4449*

**Fateme Zare**

*Reproductive Immunology Research Center, Shahid Sadoughi University of Medical Sciences, Yazd, Iran,*

**Maryam Imani**

*Reproductive Immunology Research Center, Shahid Sadoughi University of Medical Sciences, Yazd, Iran,*

**Serajeddin Vahidi**

*Research and Clinical Center for Infertility, Yazd Reproductive Sciences Institute, Shahid Sadoughi University of Medical Sciences, Yazd, Iran.*

**Hossein Ansariniya**

*Reproductive Immunology Research Center, Shahid Sadoughi University of Medical Sciences, Yazd, Iran, 0000-0002-5731-4612*

**Ali ZareHoroki**

*Research and Clinical Center for Infertility, Yazd Reproductive Sciences Institute, Shahid Sadoughi University of Medical Sciences, Yazd, Iran.*

***Corresponding author**:

**Hossein Hadinedoushan***

*Reproductive Immunology Research Center, Shahid Sadoughi University of Medical Sciences, Yazd, Iran, Orcid ID: 0000-0002-7102-3045, Email: hhadin2000@gmail.com,* ***Tel:*** *+98 353 6285406,* ***Postcode:*** *891618863*


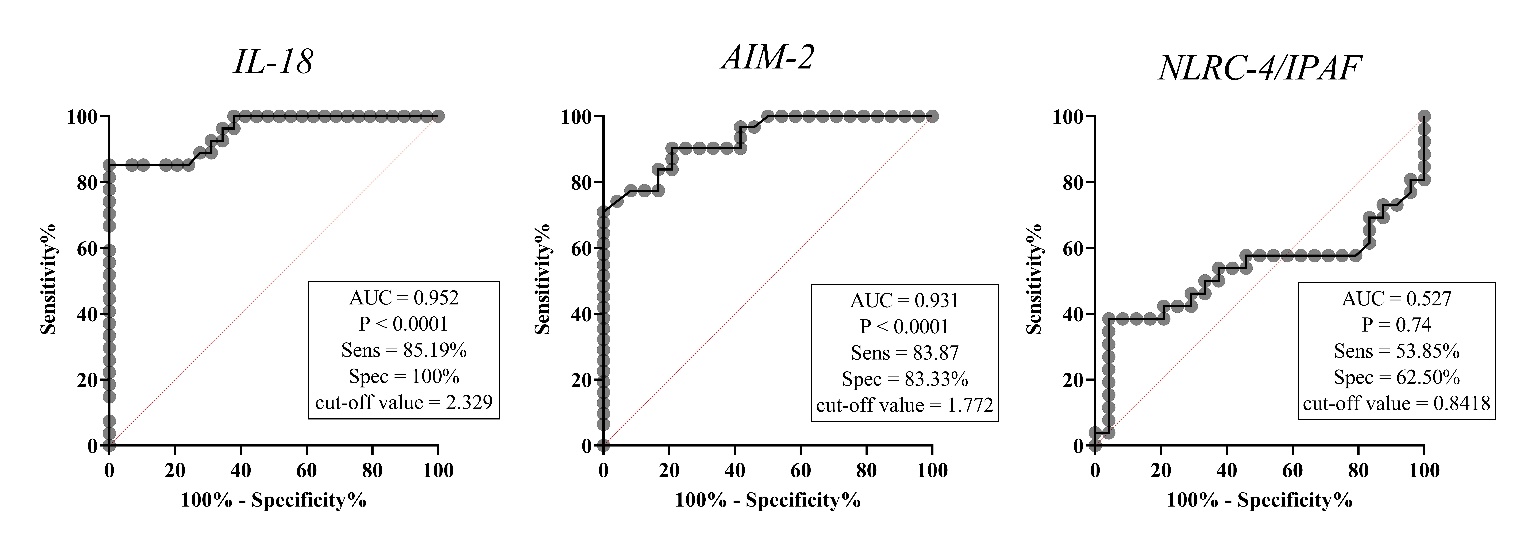


**Fig 1.** Receiver operating characteristic curve of *IL-18, AIM-2* mRNA levels in tissue samples. *IL-18; interleukin-18, AIM-2;* absent in melanoma *2, NLRC4/IPAF; NLR family CARD domain-containing protein 4/ ice* protease-activating factor. A. ROC curve comparing NOA and NS in blood samples, B. ROC curve comparing NOA and OA in blood samples. P < 0.05 is regarded as a significant value. AUC; area under curve, Sens; sensitivity, Spec; specificity.


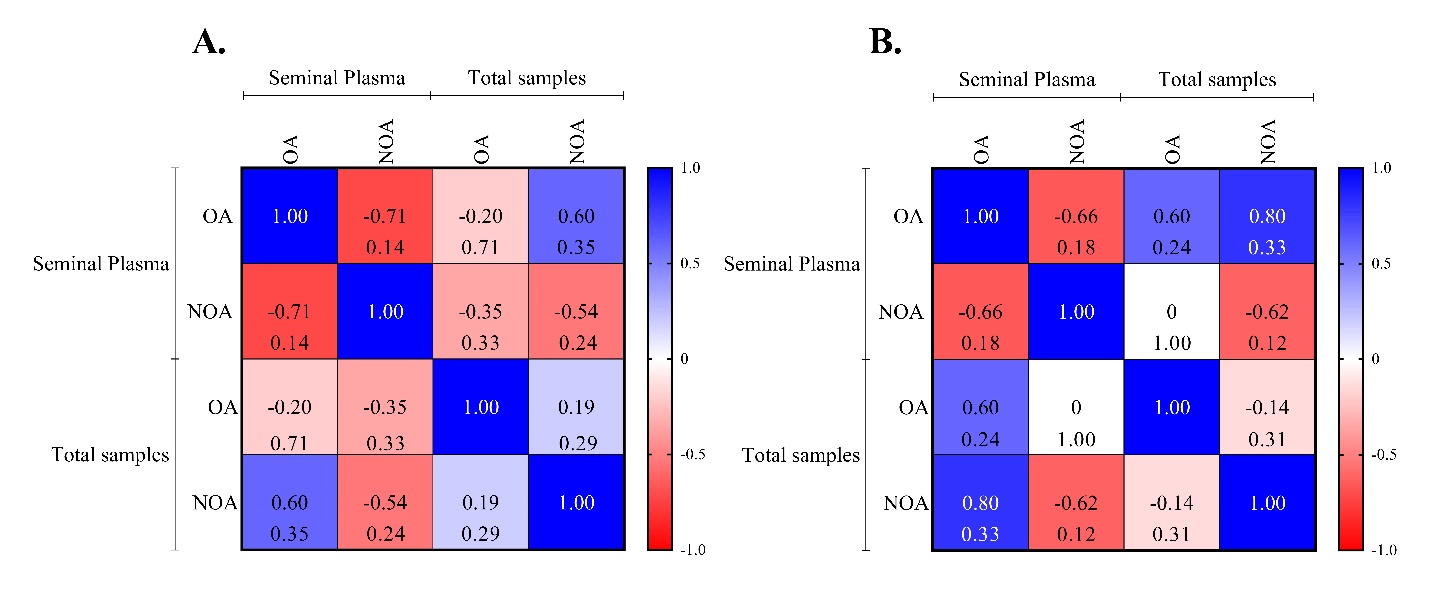


**Fig 2.** Correlation between *IL-1β* and *IL-18* in total samples and seminal plasma of both groups. OA; obstructive azoospermia, NOA; non-obstructive azoospermia *IL-1β; interleukin-1β, IL-18; interleukin-18*. In each cell, the top value is r, and the bottom is the P-value. P < 0.05 is regarded as a significant value. Where P-values are significant, numbers are written in bold and marked by an asterisk. A. heatmap of *IL-1β,* B. heatmap of *IL-18.*


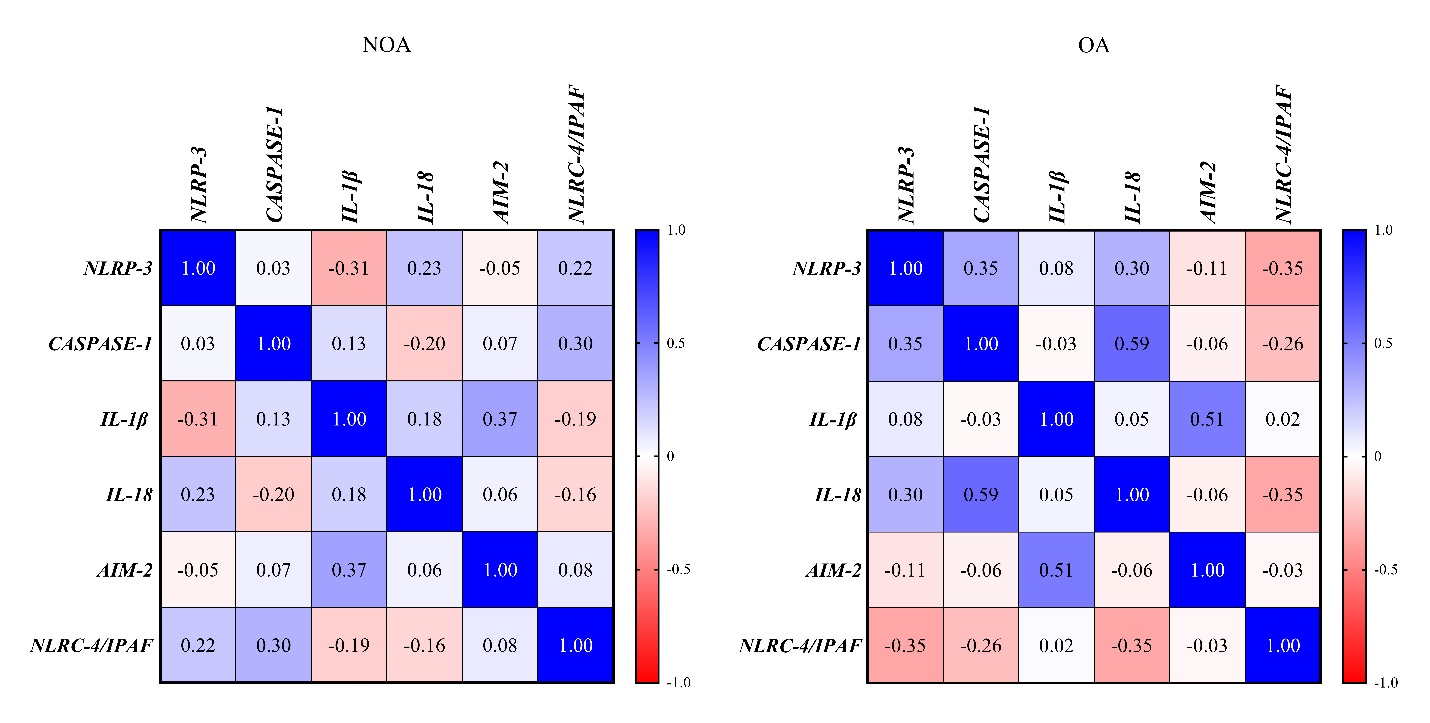


**Fig 3.** Correlation between target genes in blood samples of both groups. OA; obstructive azoospermia, NOA; non-obstructive azoospermia*, NLRP-3; NLR family pyrin domain containing 3, IL-1β; interleukin-1β,* *IL-18; interleukin-18, AIM-2;* absent in melanoma *2, NLRC4/IPAF; NLR family CARD domain-containing protein 4/ ice* protease-activating factor. In each cell, the top value is r, and the bottom is the P-value. P < 0.05 is regarded as a significant value. Where P-values are significant, numbers are written in bold and marked by an asterisk.


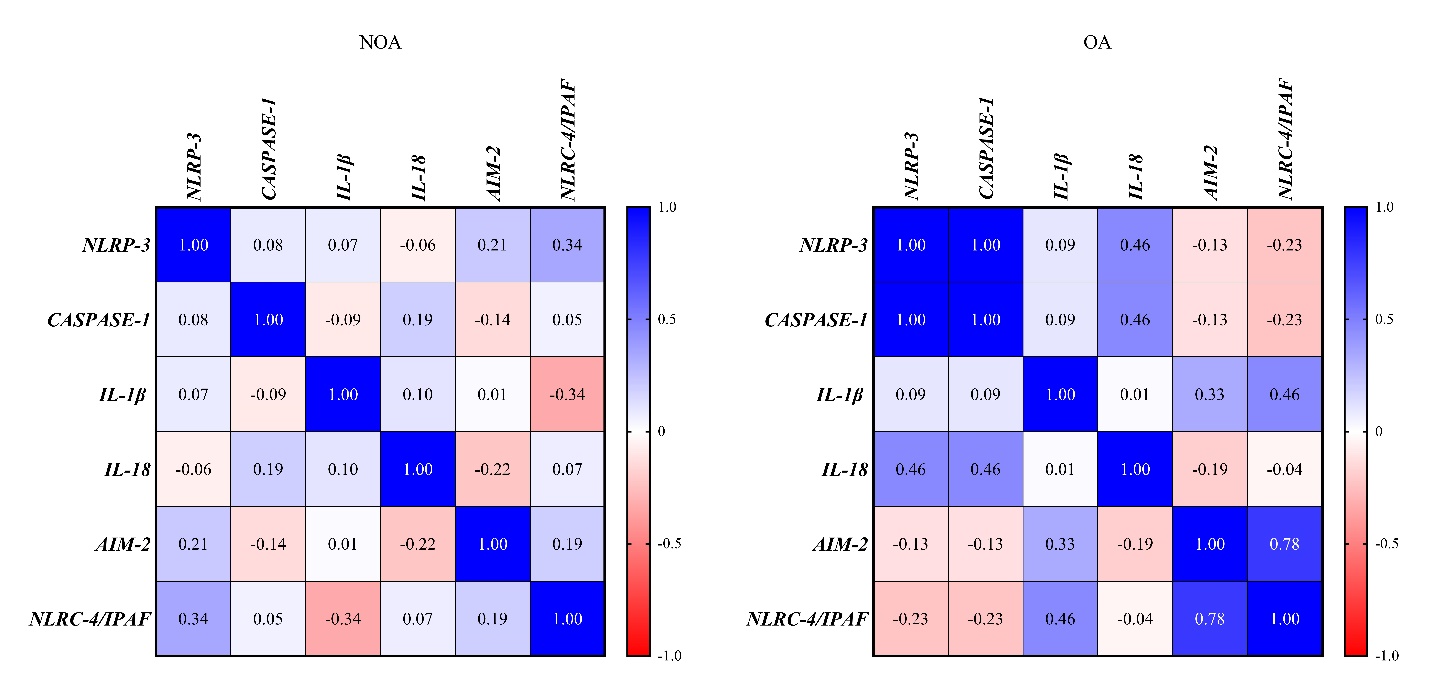


**Fig 4.** Correlation between target genes in tissue samples of both groups. OA; obstructive azoospermia, NOA; non-obstructive azoospermia*, NLRP-3; NLR family pyrin domain containing 3, IL-1β; interleukin-1β, IL-18; interleukin-18, AIM-2;* absent in melanoma *2, NLRC4/IPAF; NLR family CARD domain-containing protein 4/ ice* protease-activating factor. In each cell, the top value is r, and the bottom is the P-value. P < 0.05 is regarded as a significant value. Where P-values are significant, numbers are written bold and marked by an asterisk.
